# Intergenic Interactions of *SBNO1*, *NFAT5* and *GLT8D1* Determine the Susceptibility to Knee Osteoarthritis among Europeans of Russia

**DOI:** 10.3390/life13020405

**Published:** 2023-02-01

**Authors:** Vitaly Novakov, Olga Novakova, Maria Churnosova, Inna Sorokina, Inna Aristova, Alexey Polonikov, Evgeny Reshetnikov, Mikhail Churnosov

**Affiliations:** 1 Department of Medical Biological Disciplines, Belgorod State National Research University, 308015 Belgorod, Russia; 2 Department of Biology, Medical Genetics and Ecology and Research Institute for Genetic and Molecular Epidemiology, Kursk State Medical University, 305041 Kursk, Russia

**Keywords:** GWAS candidate genes, knee osteoarthritis, SNP, association

## Abstract

This study was conducted to examine the associations between genome-wide association studies (GWAS)-important single nucleotide polymorphisms (SNPs) and knee osteoarthritis (KOA) among Europeans of Russia. The present replicative study (“patient-control” design has been used) was carried out on 1000 DNA samples from KOA (*n* = 500) and KOA-free (*n* = 500) participants. Ten GWAS-important for KOA SNPs of eight candidate genes (*LYPLAL1, GNL3, GLT8D1, SBNO1, WWP2, NFAT5, TGFA, GDF5*) were studied. To assess the link between SNPs and KOA susceptibility, logistic regression (to establish independent SNP effects) and MB-MDR (to identify SNP–SNP interactions) were used. As a result of this genetic analysis, the associations of individual SNPs with KOA have not been proven. Eight loci out of ten tested SNPs interacted with each other (within twelve genetic models) and determined susceptibility to KOA. The greatest contribution to the disease development were made by three polymorphisms/genes such as rs6976 (C>T) *GLT8D1*, rs56116847 (G>A) *SBNO1*, rs6499244 (T>A) *NFAT5* (each was included in 2/3 [8 out 12] KOA-responsible genetic interaction models). A two-locus epistatic interaction of rs56116847 (G >A) *SBNO1* × rs6499244 (T>A) *NFAT5* determined the maximum percentage (0.86%) of KOA entropy. KOA-associated SNPs are regulatory polymorphisms that affect the expression/splicing level, epigenetic modification of 72 genes in KOA-pathogenetically significant organs such as skeletal muscles, tibial arteries/nerves, thyroid, adipose tissue, etc. These putative KOA-effector genes are mainly involved in the organization/activity of the exoribonuclease complex and antigen processing/presentation pathways. In conclusion, KOA susceptibility among Europeans of Russia is mediated by intergenic interactions (but not the main effects) of GWAS-important SNPs.

## 1. Introduction

Osteoarthritis (OA) is a common chronic degenerative joint disease [1], resulting in pathological changes in all joint tissues (cartilage/subchondral bone/ligaments/menisci/articular capsule/synovial membrane) [2]. More than 500 million people worldwide suffer from this disease; an especially high incidence of OA is observed among people over 65 years of age [3]. The prevalence of large-joint OA (knee and hip) worldwide is 3754.2 per 100 thousand population [4]. At the same time, knee joints are affected more often among the large joints [5,6]. The economic costs associated with OA are high, starting with the direct costs of care and treatment, and ending with the costs of total endoprosthetics [7]. Patients with knee OA (KOA) spend on average about USD 15,000 during their lifetime on direct medical expenses related to the disease [8]. At the same time, expensive endoprosthesis is not always effective; about 20% of patients report dissatisfaction with the result after total knee arthroplasty [9]. It is worth noting that OA leads to a decrease in the quality of life of patients through restriction of daily activity [10,11] and disability [12].

The genetic architecture of OA (including KOA) is complex and affects the individual’s total risk of developing the disorder, its clinical course, progression and prognosis [13,14,15]. The share of the hereditary component in OA is about 50% [13,14]. As a result of 24 GWAS conducted so far, 124 SNPs of 95 candidate genes associated with OA at various locations (KOA, hip joint OA, arm joint OA) have been established [15], among which 87 SNPs are linked with KOA at the genome-wide level (*p* < 5 × 10^−8^) [16]. At the same time, despite the large array of GWAS data for KOA, the number of replication studies performed is quite limited [17,18,19,20,21,22,23]. As a result of these replicative studies, associations with KOA were confirmed only for single GWAS-important SNPs: rs143383 *GDF5*, rs4867568 *LOC105374701*, rs3884606 *FGF18*, rs10947262 *BTNL2/TSBP1-AS1*, and rs7639618 *COL6A4P1*. Notably, the link with KOA in Asian populations was confirmed for three SNPs (rs143383 *GDF5*, rs4867568 *LOC105374701*, and rs3884606 *FGF18*) [22,23], and in European populations or mixed samples of Europeans and Asians for only two SNPs (rs10947262 *BTNL2/TSBP1-AS1*, rs7639618 *COL6A4P1*) [17,18].

Thus, there is an evident shortage of replicative studies of KOA. This dictates the necessity for further genetic studies of KOA in different ethnic groups of the world, including the Russian region. Therefore, we conducted this replication study of the involvement of GWAS-important SNPs for KOA disease susceptibility among Europeans of Russia.

## 2. Materials and Methods

### 2.1. Study Participants

The present replicative study (“patient-control” design has been used) was carried out on 1000 participants with KOA (*n* = 500) and KOA-free (*n* = 500). All participants (patients and control) included in the study met the following criteria (inclusion criteria): (1) individuals of European origin (all subjects had Russian nationality); (2) born and living in the central part of Russia [24,25]; (3) not related to each other; (4) aged 40 years and older; (5) the consent of all subjects (signed personally); (6) underwent the necessary clinical/laboratory/instrumental examinations in the “Orthopedics and Traumatology Department” at “Belgorod City Hospital №2” by competent orthopedic traumatologists. The following criteria were used to exclude subjects from this study (exclusion criteria): the presence of severe diabetes mellitus, coronary heart disease, hypertension, systemic connective tissue disorder, oncological diseases, renal-hepatic insufficiency, joint injuries/inflammatory, or musculoskeletal congenital malformations [26]. The sample was formed in the period between February 2016–December 2018 under the control of the hospital ethical commission.

The KOA diagnosis was established in accordance with the criteria of the ACR (American College of Rheumatology) [27,28]. The KOA group included patients with primary knee joint OA, who had a radiological stage of disease (Kellgren–Lawrence, K/L parameter) ≥ 2 [29] and pain symptoms (Visual Analog Scale [VAS] parameter was >40 points) [30]. The diagnostics of KOA was carried out by certified orthopedic traumatologists based on the results of all necessary tests (clinical/laboratory/instrumental [27,28,29,30]) conducted for all participants in the “Orthopedics and Traumatology Department” at “Belgorod City Hospital №2”. The analysis of all X-ray images was carried out by the orthopedic traumatologist Vitaly Novakov together (in consultation) with another qualified orthopedic traumatologist, which minimized the effect of bias among different observers and improved the quality of verification of the diagnosis of KOA (with r = 0.83 when reading X-ray images of patients with KOA stages 2–4 by one doctor or different doctors to r = 0.86–0.87 when reading the same X-rays by two doctors, one of whom is a permanent observer) [29]. The KOA-free group included subjects without musculoskeletal disorders. Hereditary burden (the presence of KOA and OA of other locations in relatives of 1–2 degrees of kinship, such as parents, siblings, uncles/aunts, grandparents) was assessed during a survey of the subjects (cases and controls).

### 2.2. GWAS SNPs Selection and Detection

For the present replicative study, GWAS SNPs for KOA (data from previously performed GWAS were taken into account [31,32,33,34,35,36]) (Supplementary Materials Table S1) possessing a distinct regulatory potential [37,38] (Haploreg data from the Roadmap Epigenomics and ENCODE projects were taken into account [39]) (Supplementary Table S2) were selected. As a result, we investigated ten KOA candidate SNPs: rs1060105 (C>T) and rs56116847 (G>A) *SBNO1*, rs2820436 (C>A) and rs2820443 (T>C) *LYPLAL1*, rs11177 (G>A) *GNL3*, rs6976 (C>T) *GLT8D1*, rs34195470 (G>A) *WWP2*, rs6499244 (T>A) *NFAT5*, rs3771501 (G>A) *TGFA* and rs143384 (A>G) *GDF5*.

The blood of KOA/KOA-free individuals was collected invasively from the elbow vein of the subjects and subsequently used for DNA allocation (the protocol for receiving DNA was set out earlier [40]). The NanoDrop spectrophotometer was employed to verify the DNA concentration/cleanliness [41]. DNA was stored in the freezer at −60 °C and subsequently utilized for the individual’s genotypic detection by real-time PCR (using CFX96 apparatus) [42,43]. Genotyping of DNA specimens was executed blindly by laboratory staff, and its accuracy was checked based on re-genotypic of several samples (≈7–10% case/control) [44,45], which showed a degree of 99% agreement.

### 2.3. Statistical Analysis

There was calculated the agreement of SNP genotype observed frequencies (KOA/KOA-free cohorts) to HWE [46,47]. To investigate the statistical dependence between individual SNP variants (allelic, additive, recessive, and dominant models have been employed [48]) and SNP interactions (simulation epistatic models were executed [49] with KOA using gPLINK [50] and MB-MDR [51,52] programs taking into account a number of confounders such as age, BMI, sex, hereditary, occupation-associated physical workload, leisure time physical activity, and the concomitant pathology availability (the musculoskeletal, cardiovascular, and endocrine systems) (Table 1). Parameters OR and 95% CI (odd ratio and confidence intervals of OR, respectively) have been utilized for the SNP-KOA dependence strength estimates [53,54]. Permutation-corrected *p* values were used [55] such as p_perm_ ≤ 0.0125 for individual SNPs (Bonferroni-corrected *p* value based on four calculated models was calculated additionally, 0.05/4 [56]) and p_perm_ ≤ 0.05 for models of SNP interactions [57]. Importantly, amongst the SNP epistatic models for further permutation assessment were the several models appropriate to the Bonferroni-corrected *p* value (the recombination of ten reported loci was taken into consideration) such as ≤1.11 × 10^−3^ (0.05/45 in 2–locus models), ≤4.17 × 10^−4^ (0.05/120 in 3–locus) and ≤2.38 × 10^−4^ (0.05/210 in 4–locus) [58]. The interaction map of high-confidence KOA effector SNPs was made with the MDR program [59].

### 2.4. SNPs/Genes Predict Functions

The probable KOA effector SNPs/genes (taking into account proxy SNPs, r^2^ ≥ 0.80 [60,61]) have been chosen for their functionality (the in silico approach was applied [62,63] based on online integrated resources of bioinformatics information [64,65,66] including the GTEx [67], PolyPhen2 [68], HaploReg [39], Blood eQTL [69], SIFT [70], GeneMANIA [71]).

## 3. Results

The phenotypic characteristics (common biological parameters, occupation-related/leisure time physical activity, alcohol/smoking habits, hereditary burden, and concomitant pathology) of the examined KOA/control groups are presented in Table 1. The participants’ ages ranged from 40 to 70 years in the KOA group and 40–68 years in the KOA-free (control) group. The KOA patients did not differ from the control group in age, sex, height, smoking or alcohol status (*p* > 0.05). At the same time, KOA patients had a higher BMI (*p* < 1 × 10^−6^), hereditary burden (*p* = 0.0005), obesity rate (*p* = 0.0005), and high incidence of the cardiovascular (*p* = 0.0005), endocrine (*p* = 0.02) and musculoskeletal (*p* = 0.0005) diseases compared to the control group (Table 1). In the KOA cohort, the individuals number with a high level of occupational physical activity, as well as with low physical activity in their free time, is significantly higher compared to the control group (*p* = 0.0005 and *p* = 0.0005, respectively). In turn, the control subjects registered a greater number of individuals with irregular (1.24 times) and regular (2.33 times) physical activity in their free time compared with the KOA patients (*p* = 0.04 and *p* = 0.0007, respectively) (Table 1). Additionally, in the control group, the percentage of individuals with low occupational physical activity was significantly higher (2.12 times) than in the KOA subjects (*p* = 0.0005). The above parameters were used as factor-confounders in the evaluation of SNP-disease associations.

The statistical calculations showed that all examined GWAS loci in KOA/KOA-free participants were HWE-concordant (p_HWE_ ≥ 0.117 in the KOA group and p_HWE_ ≥ 0.059 in the KOA-free cohort) (Supplementary Table S3). As a result of the genetic analysis, associations of individual SNPs with KOA have not been proven (p_bonf_ > 0.0125) (Table 2).

The present MB-MDR analysis indicated intergenic interaction of the eight GWAS loci of 10 examined SNPs (with the exclusion of rs3771501 (G>A) *TGFA* and rs143384 (A>G) *GDF5*) within twelve genetic (epistatic) models as KOA-risk/protective factors (Table 3). The greatest contribution to the disease development has been made by three polymorphisms/genes: rs6976 (C>T) *GLT8D1*, rs56116847 (G>A) *SBNO1*, and rs6499244 (T>A) *NFAT5* (each was included in 2/3 [8 out 12] KOA-responsible genetic interaction models). The highest Wald index (this statistic for high risk category equaled 47.56) was in a four–level genetic model: rs6499244 (T>A) *NFAT5 ×* rs56116847 (G>A) *SBNO1 ×* rs6976 (C>T) *GLT8D1 ×* rs2820443 (T>C) *LYPLAL1* (Table 3). Very important protective potential (−1.07 < *beta* value < −1.60 and *p* = 0.00003–0.00009) was found for some SNPs genotypic combinations such as TT(rs6499244)*NFAT5 ×* AG(rs56116847)*SBNO1 ×* CT(rs6976)*GLT8D1,* TT(rs6499244)*NFAT5* x AG(rs56116847)*SBNO1 ×* AG(rs11177)*GNL3*, TT(rs6499244)*NFAT5 ×* AG(rs56116847)*SBNO1 ×* CT(rs6976)*GLT8D1* xTT(rs2820443)*LYPLAL1*, TT(rs6499244)*NFAT5 ×* AG(rs56116847)*SBNO1 ×* AG(rs11177)*GNL3 ×* TT(rs2820443)*LYPLAL1*, TT(rs6499244)*NFAT5 ×* AG(rs56116847)*SBNO1 ×* AG(rs11177)*GNL3 ×* CT(rs6976)*GLT8D1* (Supplementary Table S4).

**Figure 1:**
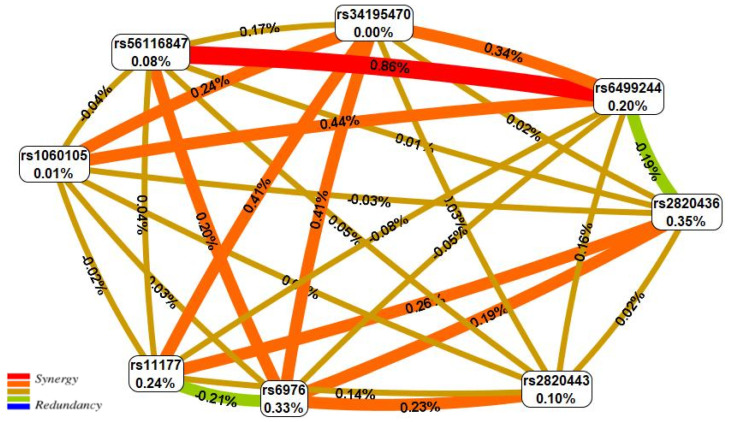
The entropy graph of the SNP × SNP interactions with knee osteoarthritis based on the MDR analysis. Positive values of entropy indicate synergistic interactions while the negative values indicate redundancy. The red and orange colors denote strong and moderate synergism, respectively, brown color denotes the independent effect, green and blue colors denote moderate and strong antagonism, respectively.

Figure 1 portrays the interaction map for eight high-confidence KOA effector SNPs. The two-locus synergetic interaction (plotted in red) of rs56116847 (G>A) *SBNO1* and rs6499244 (T>A) *NFAT5* possessed a strong KOA effect (registering the maximum KOA entropy percentage-0.86%).

### 3.1. Functional Genomics Data for KOA-Involved SNPs

#### 3.1.1. SNPs Correlations with Amino Acid Replacements and Epigenetic Changes

Five of the investigated 315 SNPs (8 KOA-causal variants and 309 proxy SNPs) were missense loci leading to amino acid substitutions in the GNL3 (Arg39Gln, rs11177 and Val367Met, rs2289247), SPCS1 (Pro41Ala, rs6617), NEC4 (Pro225Ala, rs1029871) and SBNO1 (Ser728Asn, rs1060105) proteins. The above changes were predicted to be tolerated (SIFT tools information) and benign (PolyPhen-2 resource material), and only Pro>Ala replacement at position 225 in NEC4 protein (rs1029871) was possibly damaging (prediction effect revealed by PolyPhen-2)) (Supplementary Table S5). 

Epigenetic modification of DNA regions near eighteen genes (C3orf78, CDK2AP1, CTD-2033A16.3, GLT8D1, GNL3, ITIH1, NEK4, NFAT5, NQO1, PBRM1, RN5S375, RP11-95P13.1, RP11-95P13.2, RP13-942N8.1, SBNO1, SETD8, SPCS1, WWP2) can be caused by eight KOA-involved SNPs and 292 loci out of 309 in LD SNPs (94.50%) due to the regulation of the activity of enhancers (139 SNPs, 44.13%) and promoters (24 SNPs, 7.62%), communication of specific DNA sites with protein-regulators (25 SNPs, 7.94%) and factors that promote the transcription process (266 SNPs, 86.08%) by changing the structure of chromatin (transformations of “open-closed” chromatin sites, methylation and acetylation of some histone protein fractions [H3K4me1,H3K27ac, etc.], etc.) (Supplementary Table S6). 

It is important to underline that almost all KOA-associated polymorphic variants (with the exception of rs1060105) and a majority of strongly-linked loci exhibit their outstanding epigenetic effects in KOA-impact cell cultures and tissues such as primary osteoblast cells, chondrocytes, adipocytes, etc. For example, SNPs of genes such as *LYPLAL1* (rs2820436 and rs2820443), *GNL3* (rs11177), *GLT8D1* (rs6976), *WWP2* (rs34195470) and *NFAT5* (rs6499244) were localized in the enhancer DNA regions at chondrocyte cell culture (E049(Epigenome ID)/STRM.CHON.MRW.DR.MSC(Mnemonic)), primary osteoblast cells (E129(Epigenome ID)/BONE.OSTEO(Mnemonic)); loci of *LYPLAL1* (rs2820443), *GNL3* (rs11177) and *GLT8D1* (rs6976) genes have been positioned in the adipocytes promoter sites (E023(Epigenome ID)/FAT.MSC.DR.ADIP(Mnemonic)); specific nucleotide changes of *GNL3* (rs11177), *GLT8D1* (rs6976) and *SBNO1* (rs56116847) genes were situated in the enhancer positions of peripheral blood T helper cells (E043(Epigenome ID)/BLD.CD4.CD25M.TPC(Mnemonic)).

#### 3.1.2. KOA-Associated SNPs as Gene Quantitative Traits (eQTL and sQTL) Potential Predictors

The four KOA-associated loci (rs1060105 *SBNO1*, rs11177 *GNL3*, rs6499244 *NFAT5*, rs6976 *GLT8D1*) (Supplementary Table S7) and 70 highly-coupled SNPs with them (Supplementary Table S8) were modulators of blood transcribing six genes *mRNA* level (*DNAH1, ITIH4, NT5DC2, RILPL2, SPCS1, WWP2*).

Changes in the gene quantitative traits (transcription and splicing) in different organs (including KOA pathophysiology-involved organs such as skeletal muscles, tibial arteries and nerves, adipose tissue, thyroid, etc.) due to the allele-specific effects of the eight studied SNPs and high-LD loci (283 out 309 SNPs [91.59%]) have been detected as potential predictors of KOA. In total, the 61 genes transcription (*ABCB9*, *ALAS1, ARL6IP4, C12orf65, CCDC62, CDK2AP1, CLEC18A, CLEC18C, COG4, DNAH1, EXOSC6, GLT8D1, GLYCTK, GLYCTK-AS1, GNL3, IL34, ITIH1, ITIH4, KMT5A, LYPLAL1-AS1, MPHOSPH9, MUSTN1, NEK4, NFAT5, NOB1, NPIPB14P, NQO1, NT5DC2, OGFOD2, PBRM1, PDXDC2P, PITPNM2, POC1A, PPM1M, PRKCD, RFT1, RILPL2, RIMKLBP2, RP11-168J18.6, RP11-282O18.3, RP11-296I10.3, RP11-392O17.1, RP11-394B2.1, RP11-394B2.5, RP11-546D6.3, RP11-894J14.2, RP11-972P1.11, RP5-1157M23.2, RP5-966M1.5, RP5-966M1.7, SBNO1, SERBP1P3, SFMBT1, SLC30A10, SMG1P7, SMIM4, SPCS1, TMEM110, WDR82, WWP2, ZC3H11B*) (Supplementary Tables S9 and S10) and 24 genes splicing (*ABCB9, C12orf65, GLT8D1, GLYCTK, GNL3, ITIH1, ITIH3, ITIH4, KMT5A, MPHOSPH9, MUSTN1, NOB1, NPIPB14P, NQO1, NT5DC2, PBRM1, PDXDC2P, PHF7, RILPL1, RP11-392O17.1, SMIM4, STAB1, TMEM110, WWP2*) (Supplementary Tables S11 and S12) may be changed due to the apparent eQTL and sQTL influences of KOA-correlated SNPs.

#### 3.1.3. Potential Interactions and Biological Pathways of KOA Putative Target Genes

**Figure 2:**
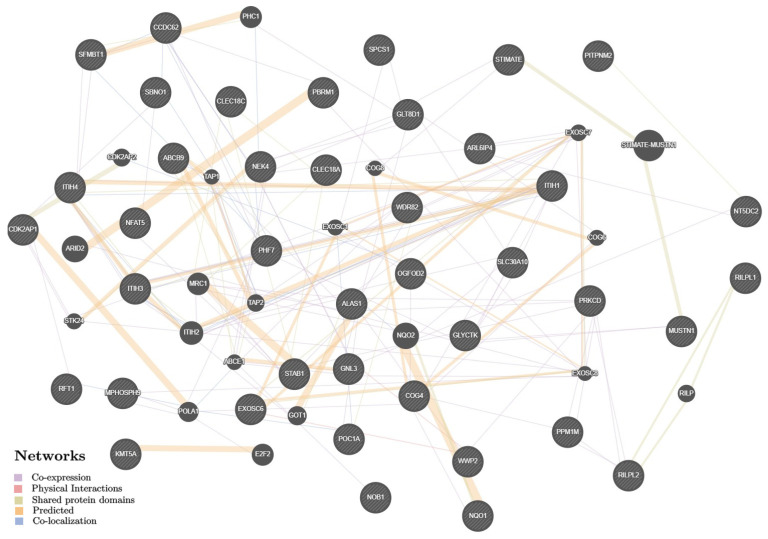
The interaction networks of 72 KOA-associated genes in various tissues/organs inferred using GeneMANIA (http://genemania.org (accessed on 15 August 2022)). The candidate genes are cross-shaded.

The 72 putative KOA-effector genes (according to the functional genomics data given above and presented in the Supplementary Tables S5–S12) have been mainly involved in the organization/activity of the exoribonuclease complex and antigen processing/presentation pathways (Supplementary Table S13), and interact among themselves through joint transcription (55.16%), predictor communications (23.26%), overall domains of proteins molecules (10.76%), joint localization (6.08%), and physical mutual influences (4.74%) (Figure 2) with a predominant potential in these relationships of prioritized interactions between the follow pairs of genes: *STIMATE-MUSTN1, STIMATE–TMEM110, STIMATE–MUSTN1, MRC1–STAB1, ARID2–PBRM1, NQO2–NQO1* (indicators of weight were 0.71–0.86) (Supplementary Table S14).

## 4. Discussion

The present “patient-control” study demonstrated that KOA susceptibility among Europeans of Russia can be caused by intergenic interactions of eight GWAS-important SNPs (rs11177 *GNL3*, rs1060105 *SBNO1*, rs2820436 and rs2820443 *LYPLAL1*, rs56116847 *SBNO1*, rs6976 *GLT8D1*, rs6499244 *NFAT5* and rs34195470 *WWP2*). The greatest contribution to the KOA susceptibility was made by the three polymorphisms/genes rs6499244 (T>A) *NFAT5,* rs6976 (C>T) *GLT8D1*, and rs56116847 (G>A) *SBNO1* (each was included in 2/3 [8 out 12] KOA-responsible genetic interaction models). The associations between individual SNPs and KOA predisposition have not been supported. 

The outcome of this work demonstrated involvement in the KOA genetic architecture (within gene-gene mutual influence) of rs6499244 (T>A) *NFAT5*. For rs6499244 *NFAT5* (located on chromosome 16q22.1), in silico data indicated its pronounced functional actions by influences on enhancer sequences in chondrocytes (E049(Epigenome ID)/STRM.CHON.MRW.DR.MSC(Mnemonic)), primary osteoblast cells (E129(Epigenome ID)/BONE.OSTEO(Mnemonic)), primary T-regulatory and T-helper cells of peripheral blood (E044(Epigenome ID)/BLD.CD4.CD25.CD127M.TREGPC(Mnemonic) and E043(Epigenome ID)/BLD.CD4.CD25M.TPC(Mnemonic), respectively), fat nuclei (E063(Epigenome ID)/FAT.ADIP.NUC(Mnemonic)), promoter sites in fat nuclei (E063(Epigenome ID)/FAT.ADIP.NUC(Mnemonic)), transcriptional levels of 10 genes (*CLEC18A, COG4, EXOSC6, IL34, NFAT5, NOB1, NPIPB14P, NQO1, PDXDC2P, SMG1P7*), and transcript alternative splicing of four genes (*NOB1, NPIPB14P, NQO1, WWP2*) in the skeletal muscles, fatty tissue, thyroid gland, and other KOA-related organs. In a previous GWAS, the causal link between rs6499244 (T>A) *NFAT5* and KOA risk was shown [35]. The potential epigenetic “authority” of this genome region was also known [15,72,73]. Rice et al. presented data on associations of the rs7359336 locus, which is in LD with rs6499244 *NFAT5* (r^2^ = 0.91), with an increased level of DNA methylation in the *WWP2* gene in patients with OA [72]. Additionally, the authors noted the connection of this functionally-active genome sequence with five cartilage-expressed genes such as *PDXDC2P, CLEC18A*, *NOB1*, *NFAT5, IL34* [72]. The *nuclear factor gene of activated T cells 5* (*NFAT5*) encodes a transcription factor that manages the quantitative expression traits of genes implicated in the regulation of osmoprotective and inflammatory reactions [73,74,75,76,77]. There is evidence of the role of *NFAT5* in the formation of innate immunity by activating gene expression in macrophages during TLR (Toll-like receptor) ligation [75,78,79]. TLR2 and TLR4 are known to be highly expressed in synovial fluid macrophages and are responsible for macrophage activation [80]. *NFAT5* also plays an important role in the proliferation of synoviocytes themselves [81]. A higher *NFAT5* expression was found in the synovial membrane [81]. *NFAT5* indirectly affects the migration of myoblasts during skeletal muscle myogenesis via the CYR61-dependent pathway [82]. Transcription factors NFAT 1, NFAT2, NFAT3 have been an essential function in the OA-pathobiology [83,84,85].

The data for rs56116847 (G>A) *SBNO1* as a KOA risk locus were first established by Tachmazidou et al. in GWAS on samples of European origin from UK Biobank and Arthritis Research UK Osteoarthritis Genetics (arcOGEN) [35]. According to our data, this locus, interacting with rs6499244 (T>A) *NFAT5*, makes the greatest contribution to KOA susceptibility (0.86% of the disease entropy) among Europeans of Russia. The literature data indicate a serious epigenetic potential (relationship with the level of methylation in cartilage) of the locus rs56116847 (G>A) *SBNO1* [15]. The information obtained by us in silico also confirms the position of this polymorphism in enhancers in primary T-regulatory and T-helper cells of peripheral blood (E044(Epigenome ID)/BLD.CD4.CD25.CD127M.TREGPC(Mnemonic) and E043(Epigenome ID)/BLD.CD4.CD25M.TPC(Mnemonic), respectively), primary peripheral blood monocytes (E029(Epigenome ID)/BLD.CD14.PC(Mnemonic)), its correlation with eQTL of eight genes (*ABCB9, ARL6IP4, C12orf65, CDK2AP1, KMT5A, MPHOSPH9, OGFOD2* and *RILPL2*) and sQTL of four genes (*ABCB9, KMT5A, MPHOSPH9* and *RILPL1*) in fibroblasts cell culture, tibial nerve, skeletal muscles, whole blood, etc. The *KMT5A* gene (also called *PR-SET7, SET8*) is closely related to the regulation of various biological processes such as DNA replication, chromosome condensation and activation of DNA replication checkpoints, cell proliferation, etc. [86]. *KMT5A* regulates the transcription of *Ras, p53* and *Wnt* genes [86], whose protein products of the same name are involved in the OA-pathophysiology [87,88,89]. For instance, *Wnt* is well known for its participation in osteogenesis due to activation of the signaling pathways such as Wnt/calcium, Wnt/cyclic adenosine monophosphate (cAMP), Wnt/c-Jun NH(2)-terminal protein kinase (JNK) and Wnt/β-catenin [87]. *ER*a plays responsible role in cartilage degradation [89]. The inhibitory importance of *KMT5A* in the proliferation and metastasis of osteosarcoma cells through the transmission of β-catenin signals is also known [90].

We have identified the most substantial contribution of the rs6976 (C>T) *GLT8D1* locus in the KOA-important intergenic interactions among Europeans of Russia. The rs6976 *GLT8D1* association with OA was shown in three previously-published GWAS in Europeans [31,32,35]. However, in two other GWAS [91,92] and one meta-analysis of nine GWAS datasets [93] of significant associations of rs6976 (C>T) *GLT8D1* with KOA in Europeans [93], African Americans [91] and Europeans of North America [92] has not been established. In the study of Lindner et al. the link between rs6976 *GLT8D1* and one of the statistical models of the shape of the proximal femur in patients with hip OA of European origin was shown [94]. In accordance with some studies, a certain shape of the proximal femur can determine the occurrence and progression of OA [95,96]. According to our in silico materials, SNP rs6976 (C>T) *GLT8D1* is functionally significant when associated with active enhancers in chondrocyte cell culture (E049(Epigenome ID)/STRM.CHON.MRW.DR.MSC(Mnemonic)), primary osteoblast cells (E129(Epigenome ID)/BONE.OSTEO(Mnemonic)), in skeletal muscle microtubule cells (E121(Epigenome ID)/MUS.HSMMT(Mnemonic)), and active promoters in adipocytes (E023(Epigenome ID)/FAT.MSC.DR.ADIP(Mnemonic)) and chondrocytes (E049(Epigenome ID)/STRM.CHON.MRW.DR.MSC(Mnemonic)). The rs6976 (C>T) *GLT8D1* locus is linked to four non-synonymous SNPs (rs11177,rs2289247,rs6617,rs1029871), which determine amino acid modification in three proteins such as GNL3 (Arg39Gln and Val367Met), SPCS1 (Pro41Ala) and NEC4 (Pro225Ala). The SNP rs6976 studied by us is associated with the expression/splicing parameters of 28 genes (*DNAH1, GLT8D1, GLYCTK, GLYCTK-AS1, GNL3, ITIH1, ITIH3, ITIH4, MUSTN1, NEK4, NT5DC2, PBRM1, PHF7, POC1A, PPM1M, RFT1, RP11-168J18.6, RP11-894J14.2, RP5-1157M23.2, RP5-966M1.5, RP5-966M1.7, SERBP1P3, SFMBT1, SMIM4, SPCS1, STAB1, TMEM110* and *WDR82*) in the tibial artery/nerve, skeletal muscles, thyroid gland, adipose tissue, etc. The data about rs6976 as a functional-impact locus were previously presented in other sources [14,97,98]. In a study by Rushton et al., rs6976 *GLT8D1* was found to be associated with a lower level of methylation in the *STAB1* gene in the cartilage of OA patients [98]. In Gee et al., the correlations of two polymorphic loci of the genes *SPCS1* (rs6617) and *GNL3* (rs11177), which are strongly coupled with rs6976 *GLT8D1*, with a significant imbalance of gene expression (*SPCS1* and *GNL3*, respectively) in cartilage and other joint tissues such as ligaments, meniscus, adipose tissue, synovial membrane, were shown [97]. A number of studies have established the association of the rs11177 (G>A) *GNL3* with the OA risk in both European and Asian populations [1,31,32,99], including on the GWAS level (*p* ≤ 5 × 10^−8^) [31,32].

The *GLT8D1* gene, located in the 3p21.1 region, encodes a protein from the glycosyltransferase family. Glycosyltransferases are a family of enzymes that catalyze the biosynthesis of oligosaccharides, polysaccharides and glycoconjugates [100]. To date, very little is known about the physiological and pathological functions of the *GLT8D1* gene and its corresponding protein. The *GNL3* gene encodes a guanine-nucleotide-binding protein (nucleostemin), which plays an important role in many processes occurring in the cell, including participation in stem cell proliferation, regulation of the cell cycle, maintenance of telomerase activity [101,102,103]. *GNL3* is expressed in mesenchymal stem cells, from which chondrocytes originate [101], shows association with chondrogenic differentiation and may participate in genomic regulation as an RNA-binding protein [104]. In a study by Louka et al., it was found that the *GNL3* gene expression in synovial tissue/fluid samples was significantly higher in the group of patients with primary OA compared with the control cohort [105]. The nucleostemin level was also increased in chondrocytes in patients with OA [31].

This study has certain limitations, which include the following: (a) only ten SNP of KOA candidate genes have been studied in the work and the inclusion of more loci in the analysis may cause a “shift” in the estimates of the intergenic interactions role of the examined loci in the formation of KOA; (b) the results of this work were not replicated on another sample from the same population or samples of another ethnic group; (c) this study did not take into account all possible risk factors for KOA (immune factors, bone metabolism, diet features, etc.) and, accordingly, their effects as covariates were not taken into account when evaluating associations; and (d) much broader experimental evidence of the functional effects of SNPs determining susceptibility to KOA is required (only in silico data were used in this work).

## 5. Conclusions

In the current study it has been revealed that the KOA susceptibility among Europeans of Russia is mediated by intergenic interactions (but not the independent effects) of GWAS-important SNPs.

## Figures and Tables

**Table 1:** Phenotypic characteristics of the study participants.

Parameters	KOA Patients $\bar{X}$ ± SD/% (*n*)	Controls $\bar{X}$ ± SD/% (*n*)	*p*
*n*	500	500	-
Men/Women	41.60/58.40 (208/292)	40.40/59.60 (202/298)	0.75
Age, years (min–max)	52.69 ± 5.67 (40–68)	52.96 ± 6.72 (40–70)	0.76
Height, cm	169.30 ± 7.89	169.90 ± 7.61	0.42
BMI, kg/m^2^	30.50 ± 5.05	26.04 ± 3.41	**<1 × 10^−06^**
Obesity (BMI ≥ 30) (yes)	51.00 (255)	13.40 (67)	**0.0005**
Alcohol (yes)	76.00 (380)	78.80 (394)	0.33
Smoker (yes)	19.00 (95)	21.00 (105)	0.48
Hereditary burden (yes)	39.00 (195)	14.60 (73)	**0.0005**
Occupation-related physical workload			
Low	18.40 (92)	39.00 (195)	**0.0005**
Medium	50.20 (251)	45.20 (226)	0.13
High	31.40 (157)	15.80 (79)	**0.000** **5**
Leisure time physical activity			
Little	69.60 (348)	56.40 (282)	**0.0005**
Irregular	25.00 (125)	31.00 (155)	**0.0** **4**
Regular	5.40 (27)	12.60 (63)	**0.0007**
Concomitant pathology			
Digestive system	12.00 (60)	10.20 (51)	0.42
Cardiovascular system	36.80 (184)	18.60 (93)	**0.0005**
Genitourinary system	5.80 (29)	5.20 (26)	0.78
Central nervous system	10.40 (52)	8.40 (42)	0.33
Musculoskeletal system	7.80 (39)	0(0)	**0.0005**
Endocrine system	10.20 (51)	6.00 (30)	**0.02**
Respiratory system	11.80 (59)	9.6 (48)	0.31
Other	5.80 (29)	5.00 (25)	0.67

Note: *p* values < 0.05 are shown in bold.

**Table 2:** Associations of the studied gene polymorphisms with knee osteoarthritis.

SNP	Gene	Minor Allele	*n*	Allelic Model				Additive Model				Dominant Model				Recessive Model			
				OR	95%CI		*p*	OR	95%CI		*p*	OR	95%CI		*p*	OR	95%CI		*p*
					L95	U95			L95	U95			L95	U95			L95	U95	
rs2820436	*LYPLAL1*	A	998	0.82	0.68	0.99	0.042	0.77	0.60	0.99	0.038	0.76	0.56	1.06	0.115	0.58	0.33	1.00	0.052
rs2820443	*LYPLAL1*	C	984	1.04	0.85	1.27	0.700	0.94	0.73	1.21	0.617	0.95	0.69	1.31	0.749	0.83	0.46	1.52	0.552
rs3771501	*TGFA*	A	997	1.06	0.88	1.26	0.552	1.00	0.80	1.24	0.968	1.01	0.72	1.40	0.965	0.96	0.66	1.44	0.902
rs11177	*GNL3*	A	1000	0.86	0.71	1.02	0.073	0.78	0.62	0.98	0.031	0.71	0.50	0.99	0.046	0.74	0.50	1.10	0.135
rs6976	*GLT8D1*	T	962	0.84	0.70	1.00	0.049	0.77	0.62	0.97	0.026	0.70	0.47	0.95	0.024	0.76	0.51	1.13	0.172
rs1060105	*SBNO1*	T	1000	1.04	0.84	1.29	0.703	1.18	0.91	1.54	0.202	1.14	0.82	1.56	0.444	1.75	0.91	3.35	0.092
rs56116847	*SBNO1*	A	998	0.93	0.77	1.11	0.414	0.86	0.68	1.08	0.200	0.84	0.61	1.16	0.296	0.77	0.47	1.24	0.283
rs6499244	*NFAT5*	A	999	1.02	0.86	1.22	0.790	1.13	0.91	1.41	0.269	1.29	0.91	1.82	0.156	1.07	0.73	1.56	0.730
rs34195470	*WWP2*	A	996	1.00	0.84	1.20	0.997	1.12	0.89	1.41	0.340	1.11	0.78	1.59	0.559	1.21	0.82	1.79	0.326
rs143384	*GDF5*	G	998	1.13	0.95	1.35	0.165	1.20	0.96	1.03	0.101	1.41	1.00	1.97	0.049	1.14	0.77	1.69	0.524

Note: All results were obtained after adjustment for covariates; OR: odds ratio; 95% CI: 95% confidence interval.

**Table 3:** SNP *×* SNP interactions significantly associated with knee osteoarthritis.

N	SNP *×* SNP Interaction Models	NH	*beta*H	WH	NL	*beta*L	WL	p_perm_
Two-order interaction models (*p* < 9.25 × 10^−04^)								
1	rs11177 *GNL3 ×* rs2820436 *LYPLAL1*	1	0.418	4.88	2	−0.060	12.20	0.007
2	rs6499244 *NFAT5 ×* rs56116847 *SBNO1*	0	-	-	2	−0.574	11.98	0.008
3	rs34195470 *WWP2 ×* rs6976 *GLT8D1*	3	0.561	13.33	1	0.786	4.98	0.010
4	rs6976 *GLT8D1 ×* rs2820443 *LYPLAL1*	3	0.567	11.50	1	−0.475	5.18	0.014
5	rs6976 *GLT8D1 ×* rs2820436 *LYPLAL1*	1	0.467	5.85	2	−0.989	10.97	0.018
Three-order interaction models (*p* < 2.71 × 10^−11^)								
1	rs6499244 *NFAT5 ×* rs56116847 *SBNO1 ×* rs6976 *GLT8D1*	1	0.558	4.12	5	−1.279	44.38	<0.001
2	rs6499244 *NFAT5 ×* rs56116847 *SBNO1 ×* rs11177 *GNL3*	2	0.670	9.04	5	−1.198	39.69	<0.001
Four-order interaction models (*p* < 5.34 × 10^−12^)								
1	rs6499244 *NFAT5 ×* rs56116847 *SBNO1 ×* rs6976 *GLT8D1 ×* rs2820443 *LYPLAL1*	3	0.927	15.13	7	−1.580	47.56	<0.001
2	rs6499244 *NFAT5 ×* rs56116847 *SBNO1 ×* rs1060105 *SBNO1 ×* rs6976 *GLT8D1*	4	1.187	16.97	7	−1.377	43.43	<0.001
3	rs6499244 *NFAT5 ×* rs56116847 *SBNO1 ×* rs11177 *GNL3 ×* rs2820443 *LYPLAL1*	5	1.179	30.55	7	−1.475	43.00	<0.001
4	rs6499244 *NFAT5 ×* rs34195470 *WWP2 ×* rs56116847 *SBNO1 ×* rs6976 *GLT8D1*	5	1.289	24.91	7	−1.518	42.00	<0.001
5	rs6499244 *NFAT5 ×* rs56116847 *SBNO1 ×* rs11177 *GNL3 ×* rs6976 *GLT8D1*	2	0.670	9.04	5	−1.257	41.00	<0.001

Note: The results were obtained using the MB-MDR method with adjustment for covariates; NH: number of significant high risk genotypes in the interaction; *beta* H: regression coefficient for high risk exposition in the step2 analysis; WH: Wald statistic for high risk category; NL: number of significant low risk genotypes in the interaction; *beta* L: regression coefficient for low risk exposition in the step2 analysis; WL: Wald statistic for low risk category; p_perm_: permutation *p*-value for the interaction model (1000 permutations).

## Data Availability

The data generated in the present study are available from the corresponding author upon reasonable request.

## Supplementary Materials

The following supporting information can be downloaded at: https://www.mdpi.com/article/10.3390/life13020405/s1, Table S1: The literature data about associations of the studied polymorphisms of the candidate genes with osteoarthritis; Table S2: The regulatory potential of the studied SNPs; Table S3: The allele and genotype frequencies of the studied SNPs in the knee osteoarthritis and control groups; Table S4: Genotype combinations associated with knee osteoarthritis; Table S5: Non-synonymous KOA-associated SNPs and SNPs in high LD (r^2^ ≥ 0.80); Table S6: Regulatory effects of the eight KOA-associated SNPs of the candidate genes and SNPs in high LD (r^2^ ≥ 0.80); Table S7: Effect of the KOA-associated candidate genes polymorphisms on affinity of the DNA regulatory motifs; Table S8: Cis-eQTL values of the KOA-associated SNPs of the candidate genes in blood; Table S9: Cis-eQTL values of the SNPs in high LD (r^2^ ≥ 0.80) with KOA-associated SNPs of the candidate genes in blood; Table S10: eQTL values of the KOA-associated SNPs of the candidate genes; Table S11: Effect of SNPs in high LD (r^2^ ≥ 0.80) with the KOA-associated polymorphisms of the candidate genes on gene expression level; Table S12: sQTL values of the KOA-associated SNPs of the candidate genes; Table S13: Effect of SNPs in high LD (r^2^ ≥ 0.80) with the KOA-associated polymorphisms of the candidate genes on alternative splicing (cis-sQTL); Table S14: Gene set enrichment analysis of biological pathways with 72 KOA-associated genes (KOA-associated polymorphisms functionality in various tissue/organs).
