# Supplementary material for: Intergenic Interactions of SBNO1, NFAT5 and GLT8D1 Determine the Susceptibility to Knee Osteoarthritis among Europeans of Russia
Source: Life (Basel). 2023 Feb 1;13(2):405. doi: 10.3390/life13020405 (PMC9960278; doi:10.3390/life13020405)
Supplement: Supplementary file 1 [file life-13-00405-s001.zip › +-Suppl Table S1.pdf]

Supplementary Table S1

The literature data about associations of the studied polymorphisms of the candidate genes with osteoarthritis

Chr	SNP	Gene	Phenotype	Association, significance (associated allele/genotype/ haplotype)	Reference
1	rs2820436	*LYPLALI*	HOA	OR=0.93, p=9.4x10^{-09} (C)	Styrkarsdottir et al., 2018
			OA	OR=0.93, p=2.01x10^{-09} (C)	Zengini et al., 2018
			KOA and/or HOA	OR=0.95, p=2.44x10^{-09} (C)	Tachmazidou et al., 2019
1	rs2820443	*LYPLALI*	KOA and/or HOA	OR=1.06, p=6.01x10^{-11} (C)	Tachmazidou et al. 2019
2	rs3771501	*TGFA*	HOA	OR=0.93, p=1.7 x10^{-08} (G)	Styrkarsdottir et al., 2018
			OA	OR=0.94, p=1.66 x10^{-08} (G)	Zengini et al., 2018
			KOA and/or HOA	OR=0.95, p=1.84 x10^{-12} (G)	Tachmazidou et al., 2019
			HOA	OR=0.92, p=1.05 x10^{-13} (G)	Tachmazidou et al., 2019
			OA	OR=0.96, p=4.24x10^{-16} (G)	Tachmazidou et al., 2019
			Hand OA	OR=1.08, p=1.9 x10^{-10} (A)	Boer et al., 2021
			THR	OR=1.07, p=1.6 x10^{-10} (A)	Boer et al., 2021
			TJR	OR=1.06, p=8.3 x10^{-11} (A)	Boer et al., 2021
			KOA and/or HOA	OR=1.04, p=8.8 x10^{-13} (A)	Boer et al., 2021
			HOA	OR=1.07, p=6.6 x10^{-13} (A)	Boer et al., 2021
			OA	OR=1.04, p=4.05x10^{-15} (A)	Boer et al., 2021
3	rs11177	*GNL3*	OA	OR=1.09, p=5.13 x10^{-09} (A)	Zeggini et al., 2012
			TJR	OR=1.12, p=1.25 x10^{-10} (A)	Zeggini et al., 2012
			HOA	OR=1.07, p=6.5 x10^{-08} (A)	Styrkarsdottir et al., 2018
3	rs6976	*GLT8D1*	OA	OR=1.09, p=6.56 x10^{-09} (T)	Zeggini et al., 2012
			TJR	OR=1.12, p=7.24 x10^{-11} (T)	Zeggini et al., 2012
			HOA	OR=1.07, p=4.5 x10^{-08} (T)	Styrkarsdottir et al., 2018
			KOA and/or HOA	OR=1.05, p=2.04 x10^{-10} (T)	Tachmazidou et al., 2019

			HOA	OR=1.08, p=3.10 $x10^{-11}$ (T)	Tachmazidou et al., 2019
12	rs1060105	*SBNO1*	KOA	OR=1.07, p=1.9$x10^{-08}$ (C)	Styrkarsdottir et al., 2018
12	rs56116847	*SBNO1*	KOA	OR=1.06, p=3.19$x10^{-10}$ (A)	Tachmazidou et al., 2019
16	rs6499244	*NFAT5*	KOA	OR=1.06, p=3.88$x10^{-11}$ (A)	Tachmazidou et al., 2019
20	rs34195470	*WWP2*	KOA	OR=1.07, p=2.7$x10^{-11}$ (G)	Styrkarsdottir et al., 2018
			TKR	OR=0.93, p=3.2$x10^{-10}$ (A)	Boer et al., 2021
			KOA	OR=0.95, p=3.1$x10^{-13}$ (A)	Boer et al., 2021
20	rs143384	*GDF5*	OA	OR=1.05, p=2.1$x10^{-10}$(A)	Styrkarsdottir et al., 2018
			KOA	OR=1.10, p=1.4$x10^{-19}$(A)	Styrkarsdottir et al., 2018
			KOA	OR=0.91, p=4.2$x10^{-23}$ (G)	Styrkarsdottir et al., 2019
			KOA	OR=1.1, p=4.77$x10^{-23}$ (A)	Tachmazidou et al., 2019
			OA	OR=1.03, p=3.04$x10^{-11}$ (A)	Boer et al., 2021
			TJR	OR=1.06, p=5.9$x10^{-12}$ (A)	Boer et al., 2021
			TKR	OR=1.10, p=6.2$x10^{-15}$ (A)	Boer et al., 2021
			KOA and/or HOA	OR=1.06, p=1.2$x10^{-20}$ (A)	Boer et al., 2021
			KOA	OR=1.07, p=1.01$x10^{-23}$ (A)	Boer et al., 2021

Note: OA–osteoarthritis; KOA–knee osteoarthritis; HOA–hip osteoarthritis; KOA and/or HOA–knee and/or hip osteoarthritis; TKR–total knee replacement; THR–total hip replacement; TJR–total joint replacement.
