# Supplementary material for: Intergenic Interactions of SBNO1, NFAT5 and GLT8D1 Determine the Susceptibility to Knee Osteoarthritis among Europeans of Russia
Source: Life (Basel). 2023 Feb 1;13(2):405. doi: 10.3390/life13020405 (PMC9960278; doi:10.3390/life13020405)
Supplement: Supplementary file 1 [file life-13-00405-s001.zip › +-Suppl table S3.pdf]

Supplementary Table S3

The allele and genotype frequencies of the studied SNPs in the knee osteoarthritis and control groups

Chr	SNP	Gene	Minor allele	Major allele	Minor allele frequency	Number of the studied chromosomes	Genotype distribution*	H_o	H_e	P_{HWE}
Knee osteoarthritis patients (n=500)										
1	rs2820436	*LYPLAL1*	A	C	0.283	996	38/206/254	0.414	0.406	0.741
1	rs2820443	*LYPLAL1*	C	T	0.268	1000	40/188/272	0.376	0.392	0.362
2	rs3771501	*TGFA*	A	G	0.433	998	96/240/163	0.481	0.491	0.649
3	rs11177	*GNL3*	A	G	0.436	1000	97/242/161	0.484	0.492	0.717
3	rs6976	*GLT8D1*	T	C	0.436	974	97/231/159	0.474	0.492	0.461
12	rs1060105	*SBNO1*	T	C	0.219	1000	30/159/311	0.318	0.342	0.117
12	rs56116847	*SBNO1*	A	G	0.340	1000	60/220/220	0.440	0.449	0.690
16	rs6499244	*NFAT5*	A	T	0.463	998	104/254/141	0.509	0.497	0.653
16	rs34195470	*WWP2*	A	G	0.476	994	110/253/134	0.509	0.499	0.719
20	rs143384	*GDF5*	G	A	0.453	996	106/239/153	0.480	0.496	0.471
Control group (n=500)										
1	rs2820436	*LYPLAL1*	A	C	0.325	1000	56/213/231	0.426	0.439	0.541
1	rs2820443	*LYPLAL1*	C	T	0.260	968	41/170/273	0.351	0.385	0.059
2	rs3771501	*TGFA*	A	G	0.420	996	98/222/178	0.446	0.487	0.066
3	rs11177	*GNL3*	A	G	0.476	1000	113/250/137	0.500	0.499	1.000
3	rs6976	*GLT8D1*	T	C	0.481	950	111/235/129	0.495	0.499	0.854
12	rs1060105	*SBNO1*	T	C	0.212	1000	27/158/315	0.316	0.334	0.229
12	rs56116847	*SBNO1*	A	G	0.357	996	71/214/213	0.430	0.459	0.145
16	rs6499244	*NFAT5*	A	T	0.457	1000	114/229/157	0.458	0.496	0.087
16	rs34195470	*WWP2*	A	G	0.476	998	112/251/136	0.503	0.499	0.929
20	rs143384	*GDF5*	G	A	0.422	1000	97/228/175	0.456	0.488	0.143

Note: * minor allele homozygotes / heterozygotes / major allele homozygotes
