# Supplementary material for: Intergenic Interactions of SBNO1, NFAT5 and GLT8D1 Determine the Susceptibility to Knee Osteoarthritis among Europeans of Russia
Source: Life (Basel). 2023 Feb 1;13(2):405. doi: 10.3390/life13020405 (PMC9960278; doi:10.3390/life13020405)
Supplement: Supplementary file 1 [file life-13-00405-s001.zip › +-Suppl table S4.pdf]

Supplementary Table S4

Genotype combinations associated with knee osteoarthritis *

Model	N	Genotype combinations	*beta*	P	Risk. High/Low
Two-order interaction models					
1	1	GG (rs11177)xCC (rs2820436)	0.42	0.027	H
	2	AG (rs11177)xAA (rs2820436)	-1.08	0.002	L
2	3	TT (rs6499244)xAG (rs56116847)	-0.55	0.007	L
3	4	AG (rs34195470)xCC (rs6976)	0.40	0.040	H
	5	AA (rs34195470)xTT (rs6976)	-0.79	0.026	L
4	6	TT (rs6976)xTT (rs2820443)	-0.46	0.023	L
	7	TT (rs6976)xCT (rs2820443)	0.54	0.044	H
5	8	CC (rs6976)xCC (rs2820436)	0.47	0.016	H
	9	CT (rs6976)xAA (rs2820436)	-0.98	0.005	L
Three-order interaction models					
1	1	TT (rs6499244)xGG (rs56116847)xCT (rs6976)	0.56	0.042	H
	2	AA (rs6499244)xGG (rs56116847)xCT (rs6976)	-1.10	0.003	L
	**3**	**TT (rs6499244)xAG (rs56116847)xCT (rs6976)**	**-1.14**	**0.00003**	**L**
	4	TT (rs6499244)xGG (rs56116847)xTT (rs6976)	-0.81	0.044	L
	5	AT (rs6499244)xAA (rs56116847)xTT (rs6976)	-1.80	0.024	L
2	6	TT (rs6499244)xGG (rs56116847)xAG (rs11177)	0.66	0.019	H
	7	AA (rs6499244)xGG (rs56116847)xAG (rs11177)	-1.01	0.008	L
	**8**	**TT (rs6499244)xAG (rs56116847)xAG (rs11177)**	**-1.07**	**0.00009**	**L**
	9	AT (rs6499244)xAA (rs56116847)xAA (rs11177)	-1.80	0.024	L
	10	AA (rs6499244)xAA (rs56116847)xAA (rs11177)	-1.59	0.044	L
Four-order interaction models					
1	1	TT(rs6499244)xGG (rs56116847)xCT (rs6976)xTT (rs2820443)	0.91	0.015	H
	**2**	**TT(rs6499244)xAG (rs56116847)xCT (rs6976)xTT (rs2820443)**	**-1.60**	**0.00003**	**L**
	3	TT (rs6499244)xGG (rs56116847)xTT (rs6976)xTT (rs2820443)	-2.02	0.009	L
	4	AT (rs6499244)xAA (rs56116847)xTT (rs6976)xTT (rs2820443)	-1.61	0.048	L
	5	AA (rs6499244)xGG (rs56116847)xCT (rs6976)xCT (rs2820443)	-1.15	0.033	L
2	6	AA(rs6499244)xGG (rs56116847)xCC (rs1060105)xCT (rs6976)	-1.65	0.012	L
	7	TT(rs6499244)xAG (rs56116847)xCC (rs1060105)xCT (rs6976)	-0.15	0.0005	L
	8	TT(rs6499244)xGG (rs56116847)xCT (rs1060105)xCT (rs6976)	1.06	0.011	H
	9	TT(rs6499244)xAG (rs56116847)xCT (rs1060105)xCT (rs6976)	-1.01	0.032	L
3	10	TT(rs6499244)xGG (rs56116847)xAG (rs11177)xTT (rs2820443)	1.12	0.004	H
	**11**	**TT(rs6499244)xAG (rs56116847)xAG (rs11177)xTT**	**-1.58**	**0.00005**	**L**

		**(rs2820443)**			
	12	TT (rs6499244)xGG (rs56116847)xAA (rs11177)xTT (rs2820443)	-1.38	0.018	L
	13	AT (rs6499244)xAA (rs56116847)xAA (rs11177)xTT (rs2820443)	-1.61	0.048	L
	14	AA (rs6499244)xGG (rs56116847)xGG (rs11177)xCT (rs2820443)	2.99	0.005	H
	15	AT (rs6499244)x AG (rs56116847)xAA (rs11177)xCT (rs2820443)	1.20	0.021	H
4	16	TT (rs6499244)xAG (rs34195470)xGG (rs56116847)xCT (rs6976)	1.18	0.004	H
	17	AA (rs6499244)xAG (rs34195470)xGG (rs56116847)xCT (rs6976)	-2.01	0.011	L
	18	TT (rs6499244)xAG (rs34195470)xAG (rs56116847)xCT (rs6976)	-1.45	0.0003	**L**
	19	TT (rs6499244)xAG (rs34195470)xGG (rs56116847)xTT (rs6976)	-1.91	0.015	L
	20	AT (rs6499244)xGG (rs34195470)xAG (rs56116847)xCT (rs6976)	2.42	0.027	L
5	21	TT (rs6499244)xGG (rs56116847)xAG (rs11177)xCT (rs6976)	0.66	0.019	L
	22	AA (rs6499244)xGG (rs56116847)xAG (rs11177)xCT (rs6976)	-1.01	0.008	L
	**23**	**TT (rs6499244)xAG (rs56116847)xAG (rs11177)xCT (rs6976)**	**-1.11**	**0.00006**	**L**
	24	TT (rs6499244)xGG (rs56116847)xAA (rs11177)xTT (rs6976)	-0.81	0.044	L
	25	AT (rs6499244)xAA (rs56116847)xAA (rs11177)xTT (rs6976)	-1.80	0.024	L
	26	AA (rs6499244)xAA (rs56116847)xAA (rs11177)xTT (rs6976)	-1.58	0.044	L

***** Genotype combinations are derived from the interaction models obtained by the MB-MDR method and described in tables 3
